# A comprehensive assessment of caffeine’s effects on components of countermovement jump performance

**DOI:** 10.5114/biolsport.2022.107017

**Published:** 2021-07-03

**Authors:** Jozo Grgic

**Affiliations:** 1 Institute for Health and Sport (IHES), Victoria University, Melbourne, Australia

**Keywords:** Jumping performance, Ergogenic aid, Performance-enhancing effects, Stretch-shortening cycle

## Abstract

The aim of this study was to conduct a comprehensive examination of caffeine’s effects on countermovement jump (CMJ) performance. In this randomized, double-blind, crossover study, twenty-two resistance-trained men (age: 28 ± 5 years; height: 183 ± 5 cm; weight: 79 ± 10 kg; habitual caffeine intake: 127 ± 102 mg/day) performed the CMJ test on two occasions, following the ingestion of capsule containing 3 mg/kg of caffeine or placebo (3 mg/kg of dextrose). Fifteen outcomes derived from the force plate during the CMJ test were analyzed. As compared to placebo, there was a significant ergogenic effect of caffeine for peak force, force at eccentric to concentric action transition, time to peak force, peak power, maximum rate of power development, peak velocity, power at peak force, velocity at peak power, velocity at peak force, and vertical jump height. Effect sizes ranged from 0.11 to 0.38, *p*-values ranged from 0.048 to 0.002. There were no significant differences between caffeine and placebo for mean force, mean power, time to peak power, impulse at 300 ms, and force at peak power. This study shows that caffeine ingestion impacts a wide array of outcomes derived from the force plate during the CMJ test, not only jump height. From a practical perspective, the findings suggest that: (1) individuals interested in acute increases in CMJ performance may consider caffeine supplementation; and, (2) caffeine intake should be standardized before CMJ testing.

## INTRODUCTION

Caffeine is a widely consumed psychoactive stimulant [1]. A national-level survey indicated a high prevalence of caffeine intake among the general population [1]. Caffeine is also often consumed by athletes, as recent data showed that urinary caffeine concentration after competitions is detectable in the majority of athletes [2, 3]. The popularity of caffeine among athletes is likely due to its ergogenic effects on exercise performance [4]. Current evidence indicates that caffeine ingestion may enhance different components of exercise performance [4]. For example, caffeine ingestion was reported to be ergogenic for aerobic and muscle endurance, muscle strength, as well as sprint and speed performance [4].

Studies have also explored caffeine’s effects on performance in different vertical jump tasks [4–10]. Two meta-analyses pooled the data from individual studies and reported ergogenic effects of caffeine ingestion on vertical jump height [11, 12]. Despite the positive effects of caffeine on jump height, caffeine’s effects on other components of vertical jump performance remain largely unexplored. Even though jump height is certainly of importance, vertical jump performance is also described by variables such as peak force, power, velocity, time to peak force and power, impulse, rate of power development, and others [13]. Some of these variables may be of substantial practical importance. For example, countermovement jump (CMJ) variables such as mean power, time to peak power, rate of force development, and velocity at peak power are suggested to be highly sensitive for neuromuscular fatigue monitoring [14]. Furthermore, some CMJ-derived variables, such as peak power and peak velocity, are significantly correlated with maximum speed and muscular strength [13, 15]. Additionally, research demonstrated that CMJ impulse and mean power are able to discriminate between senior and academy rugby league players and different levels of elite status among handball players [16, 17]. Despite their practical importance, the effects of caffeine on these outcomes are rarely reported and analyzed in the scientific literature. Indeed, jump height is an outcome that is almost exclusively reported in previous research [4–10].

Given that several different variables evaluate overall jump performance, practitioners utilizing vertical jump tests on a force plate are likely to be interested in a wide array of outcomes, not only vertical jump height. Therefore, this study aimed to conduct a comprehensive examination of caffeine’s effects on vertical jump performance. It was hypothesized that caffeine ingestion would be acutely ergogenic for jump height and all other recorded outcomes.

## MATERIALS AND METHODS

### Design

This study utilized a double-blind, randomized, counterbalanced, and crossover design. In total, the participants went through the testing protocol four times: two familiarization sessions and two main sessions. To minimize the effect of diurnal biological variations, each participant was tested at the same time of day [18]. All testing sessions were performed in the morning hours (between 07:00 and 12:00 h). A minimum of four and a maximum of seven days separated every testing session. After the two practice sessions, the two main trials were performed. These trials included the ingestion of caffeine or placebo. In these sessions, the participants came to the testing laboratory in a fasted state (overnight fast). Caffeine and placebo were administered in a capsule form. Caffeine was provided in a dose of 3 mg per kg of body mass, while the placebo contained 3 mg/kg of dextrose. A caffeine dose of 3 mg/kg was used given that it has been previously reported to be ergogenic [7]. In both cases (i.e., caffeine and placebo trials), the capsules were ingested 60 minutes pre-exercise; this timing of ingestion is suggested to correspond to peak plasma caffeine concentrations [19]. This timing of ingestion was also used in most other studies that explored the effects of caffeine on exercise performance [5–8, 20]. In order to ensure a double-blind study design, caffeine and placebo capsules were of the same appearance and were provided to the participants in sealed envelopes. When the exercise session started, the participants first performed an upper-body resistance exercise that lasted around 30 minutes (results reported elsewhere [21, 22]). Therefore, CMJ performance was evaluated 90 minutes after ingestion of caffeine or placebo. On the days before the main sessions, the participants were advised not to perform any strenuous activity and to keep a food diary. On the days before the main sessions, the participants were also required to restrict caffeine ingestion after 6 pm.

### Participants

Resistance-trained men were included as study participants. All included participants had a minimum of six months of resistance training experience with a minimum training frequency of two days per week (on most weeks). Participants were excluded from this study if they reported: (a) use of anabolic steroids (prior or current); and/or (b) the existence of any health limitations. Power analysis was calculated using G*Power (version 3.1.9.2, University Düsseldorf, Germany). Assuming ANOVA, repeated measures, within factors as the statistical test, 0.15 as the expected effect size (*f*) for vertical jump height (determined using meta-analytical data [11]), 0.05 as α, the statistical power of 0.80, 1 group, 2 measurements, and *r* of 0.90 the power analysis indicated that the required sample size was *n* = 20. A total of 22 men (mean ± standard deviation of age: 28 ± 5 years; height: 183 ± 5 cm; weight: 79 ± 10 kg; habitual caffeine intake: 127 ± 102 mg/day) enrolled in the study, and all participants completed the testing sessions without any dropouts or any other adverse events.

Estimation of habitual caffeine intake was performed using a previously validated questionnaire [23]. Victoria University Human Research Ethics Committee provided ethical approval for this study (HRE19–019), which was registered at the Australian New Zealand Clinical Trials Registry (ID: ACTRN12619000885190). Every participant signed informed consent.

### Testing protocol

Before the CMJ test, the participants first performed a warm-up protocol that involved one minute of running followed by ten bodyweight squats. CMJ testing was performed on a force platform (400S Isotronic Fitness Technology, Skye, South Australia, Australia). The reliability of data obtained from this force platform has been reported elsewhere [24]. The participants first positioned themselves on the force platform in an upright standing position. A computer screen was set in front of the platform. On this screen, commands from the software associated with the force plate were displayed. This software first counted down “3, 2, 1” followed by “Set” and “Go” commands. Within five seconds of the “Go” command, the participants were required to perform the jump. The participants were required to perform a fast downward counter-movement to a semi-squat position followed by an “explosive” extension of the legs. Before starting with the test, the participants were instructed to jump as quickly and “explosively” as possible. A total of four jumps were performed, namely, one warm-up jump and three main attempts. Between each jump, the participants were provided with one minute of rest. For the analysis, the best values from the three jumps were used. In this study, the following outcomes were examined: peak force; mean force; force at eccentric to concentric action transition; time to peak force; peak power; mean power; maximum rate of power development; time to peak power; peak velocity; impulse at 300 ms; force at peak power; power at peak force; velocity at peak power; velocity at peak force; and vertical jump height. The description of these CMJ variables is provided in detail elsewhere [14, 25].

### Side effects

The incidence of side effects associated with caffeine and placebo supplementation was examined twice: (a) immediately after the testing sessions; and, (b) in the following mornings, upon waking. We used an 8-item survey that included a yes/no response scale for the evaluation of side effects.

### Effectiveness of blinding

The effectiveness of blinding was examined, as proposed by Saunders and colleagues [26]. Specifically, immediately before and after both main testing sessions, the participants were asked to respond to the following question: “Which supplement do you think you have ingested?”. This question had three possible answers: (a) “caffeine”, (b) “placebo” and (c) “I do not know”.

### Statistical analysis

Shapiro–Wilk test was used for evaluating the normality of distribution. After confirming normality of distribution, all CMJ performance outcomes were analyzed using one-way repeated-measures analysis of variance (ANOVA). Hedges’ g for repeated measures was used to calculate relative effect sizes (ES) and their respective 95% confidence intervals. ESs were interpreted using the following scale: < 0.20 as trivial, 0.20–0.49 as small, 0.50–0.79 as moderate, and ≥ 0.80 as large. McNemar’s test was used to explore possible differences in the incidence of side effects between caffeine and placebo conditions. The data on participants blinding were examined using Bang’s Blinding Index. These data are reported as a percentage of participants that identified the conditions beyond random chance. All analyses were performed using the Statistica software (version 13.0; StatSoft; Tulsa, OK, USA). The statistical significance threshold was set at *p* < 0.05.

## RESULTS

### Performance outcomes

As compared to placebo, there was a significant ergogenic effect of caffeine for peak force (*p* = 0.046; ES: 0.11), force at eccentric to concentric action transition (*p* = 0.041; ES: 0.14), time to peak force (*p* = 0.048; ES: 0.28), peak power (*p* = 0.020; ES: 0.16), maximum rate of power development (*p* = 0.018; ES: 0.13), peak velocity (*p* = 0.005; ES: 0.32), power at peak force (*p* = 0.038; ES: 0.38), velocity at peak power (*p* = 0.007; ES: 0.38), velocity at peak force (*p* = 0.002; ES: 0.24), and vertical jump height (*p* = 0.010; ES: 0.14). There were no significant differences (*p* > 0.05 for all) between caffeine and placebo for mean force, mean power, time to peak power, impulse at 300 ms, and force at peak power. All analyzed data are summarized in Table 1.

**TABLE 1:** Data from the force plate in the countermovement jump test.

Variable	Placebo (mean ± SD)	Caffeine (mean ± SD)	ES and its 95% CI	*p*-value
Peak force (N)	1756 ± 267	1787 ± 286	0.11 (0.00, 0.22)	0.046
Mean force (N)	793 ± 102	789 ± 97	–0.04 (–0.10, 0.02)	0.316
Force at eccentric to concentric action transition (N)	1625 ± 310	1669 ± 313	0.14 (0.00, 0.28)	0.041
Time to peak force (s)	0.75 ± 0.25	0.68 ± 0.23	0.28 (0.01, 0.57)	0.048
Peak power (W)	3791 ± 667	3902 ± 658	0.16 (0.03, 0.30)	0.020
Mean power (W)	705 ± 139	700 ± 156	–0.03 (0.33, 0.26)	0.821
Maximum rate of power development (W/s)	13633 ± 4433	14194 ± 4171	0.13 (0.01, 0.24)	0.018
Time to peak power (s)	0.28 ± 0.06	0.27 ± 0.05	0.07 (–0.05, 0.19)	0.274
Peak velocity (m/s)	2.67 ± 0.21	2.73 ± 0.19	0.32 (0.10, 0.56)	0.005
Impulse at 300 ms (Ns)	239 ± 30	238 ± 28	–0.03 (–0.10, 0.03)	0.187
Force at peak power (N)	1566 ± 254	1567 ± 259	0.00 (–0.05, 0.06)	0.924
Power at peak force (W)	3539 ± 524	3751 ± 508	0.38 (0.02, 0.78)	0.038
Velocity at peak power (m/s)	2.45 ± 0.21	2.53 ± 0.21	0.38 (0.11, 0.68)	0.007
Velocity at peak force (m/s)	2.15 ± 0.27	2.21 ± 0.24	0.24 (0.09, 0.43)	0.002
Vertical jump height (cm)	35.1 ± 5.7	35. 9 ± 5.7	0.14 (0.03, 0.25)	0.010

ES: effect size: CI: confidence interval; SD: standard deviation.

### Side effects

When assessed immediately after the testing sessions, there were significant differences only in “increased vigor/activeness” (*p* = 0.009) and “perception of improved performance” (*p* = 0.002). In both cases, the incidence of these side effects was higher in the caffeine condition. No significant differences between caffeine and placebo were found when side effects were assessed in the mornings after the testing sessions (Table 2).

**TABLE 2:** Self-perceived side effects reported immediately after testing session and in the morning after the testing session.

Variable	Placebo	Caffeine	Placebo	Caffeine
	Immediately after testing session	Immediately after testing session	Morning after testing session	Morning after testing session
Muscle soreness	27%	14%	14%	14%
Increased urine production	14%	5%	9%	0%
Tachycardia and heart palpitations	5%	5%	0%	0%
Increased anxiety	0%	14%	0%	0%
Headache	9%	9%	14%	5%
Abdominal/gut discomfort	0%	0%	0%	0%
Insomnia	n/a	n/a	0%	9%
Increased vigor/activeness	14%	64%*	0%	14%
Perception of improved performance	14%	77%*	n/a	n/a

* significant differences between conditions calculated using McNemar’s test.

### Effectiveness of blinding

When assessed before exercise, 55% and 59% of the participants correctly identified the caffeine and placebo conditions beyond random chance, respectively. When assessed immediately after exercise, 68% and 72% of the participants correctly identified the placebo and caffeine conditions beyond random chance, respectively.

## DISCUSSION

The main finding of this study is that caffeine ingestion was acutely ergogenic for vertical jump height and other outcomes derived from the force plate during the CMJ test. In addition to jump height, caffeine increased: (a) peak force and force at eccentric to concentric action transition (while decreasing the time needed to reach peak force); (b) peak power, power at peak force, and maximum rate of power development; and, (c) peak velocity, velocity at peak power, and velocity at peak force. Despite the improvements in these outcomes, no significant differences between caffeine and placebo were observed for mean force, mean power, time to peak power, impulse at 300 ms, and force at peak power.

Out of the analyzed outcomes, velocity variables seem to be particularly affected by caffeine ingestion. Indeed, all three velocity-associated outcomes (i.e., peak velocity, velocity at peak power, and velocity at peak force) improved following caffeine ingestion. ESs for these outcomes ranged from 0.24 to 0.38. In line with this finding, research that examined the effects of caffeine on other velocity-based tasks (e.g., repetition velocity in resistance exercise) also reported substantial ergogenic effects of acute caffeine ingestion [27]. Caffeine ingestion was reported to increase muscle fiber conduction velocity and motor unit recruitment, which might explain this finding from a physiological perspective [28]. To our knowledge, only two studies examined the effects of caffeine ingestion on CMJ velocity variables [25, 29]. In one of these studies, Zbinden-Foncea et al. [25] also reported that caffeine ingestion (dose of 5 mg/kg) enhanced peak velocity and velocity at peak power. However, compared to the present study, Zbinden-Foncea et al. [25] reported larger performance-enhancing effects of caffeine, as ES amounted to 0.79 and 0.90 for velocity at peak power and peak velocity, respectively. In another study [29], acute ingestion of caffeine in the dose of 3 mg/kg enhanced velocity at peak power by an ES of 0.28, which is much more similar to the ES of caffeine on velocity variables observed herein. Due to the difference in doses of caffeine used between the studies, these results might suggest that larger doses of caffeine produce greater increases in velocity outcomes in the CMJ test, even though future dose-response studies are needed to explore this topic further.

A substantial focus has been recently placed on the relationship between vertical jump height and power [30]. Even though jump height is generally not considered a good indicator of lower limb power output, increased jump height following caffeine ingestion is commonly interpreted as an increase in ‘power’ [5, 6]. Several studies that evaluated jump height and power reported ergogenic effects of caffeine on both outcomes [31, 32]. However, other studies reported that improvements in jump height occur independently of changes in power [33, 34]. In this study, caffeine affected some, but not all power variables. Specifically, following caffeine ingestion, we observed improvements in peak power, maximum rate of power development, and power at peak force. However, there was no significant difference between caffeine and placebo in mean power, suggesting that the relationship between caffeine-induced changes in vertical jump height and power is dependent on the analyzed power outcome.

While caffeine ingestion enhanced several aspects of CMJ performance, there was no significant difference between caffeine and placebo for mean force, mean power, time to peak power, impulse at 300 ms, and force at peak power. The lack of an ergogenic effect of caffeine on these outcomes might be related to their test-retest reliability. One study [35] reported that the coefficient of variation (CV) for test-retest reliability of mean power is between 7.8% and 12.3%. For time to peak power, this study reported CVs from 7.0% to 14.4%. It might be that the lack of an ergogenic effect of caffeine on these outcomes is explained by their lower test-retest reliability, which might have resulted in a type II error. This hypothesis seems plausible given that CVs for outcomes such as peak power and peak velocity are lower (1.3% to 3.3%), and for these outcomes, there was an ergogenic effect of caffeine [35].

From a practical perspective, there are two main areas where the results of this study are highly relevant. Caffeine ingestion elicited significant improvements in power, which is considered to be critical to the performance of many athletic tasks [36]. Therefore, the first practical application of the findings is that individuals interested in acute increases in power may consider supplementing with caffeine. Performance in CMJ is also reported to be correlated with speed and lower-body strength [13]. Therefore, increases in CMJ performance following caffeine ingestion may also be associated with improvements in other components of exercise performance. The second main practical application of the findings is related to the standardization of caffeine ingestion before testing. Evaluation of CMJ performance on a force platform is more likely to be used for testing than for training purposes. Therefore, our results highlight the need for standardizing caffeine ingestion before CMJ testing, especially when aiming to conduct between-group comparisons.

The main strength of this study is the inclusion of a relatively large sample size. In the two meta-analyses that examined the effects of caffeine on vertical jump height performance, the median sample size per included study was 13 and 14 participants, respectively [11, 12]. The total sample size in the present study is almost twice as large as these median values, which allowed the detection of small but potentially meaningful differences between conditions. The main limitation of this study pertains to the effectiveness of blinding. Specifically, 55% to 72% of participants correctly identified the caffeine or placebo conditions beyond random chance, respectively. This is important to mention, given that correct supplement identification may impact performance and therefore present a source of bias in sports nutrition research [26]. Additionally, the ergogenic effects presented herein are specific to a caffeine dose of 3 mg/kg and should not be generalized to the effects observed with lower or higher doses of caffeine.

## CONCLUSIONS

This study found that caffeine ingestion was acutely ergogenic for peak force, force at eccentric to concentric action transition, time to peak force, peak power, maximum rate of power development, peak velocity, power at peak force, velocity at peak power, velocity at peak force, and vertical jump height in the CMJ test. From a practical perspective, the findings suggest that: (a) individuals interested in acute increases in CMJ performance may consider supplementing with caffeine; and, (b) caffeine intake should be standardized before CMJ testing.

## Conflict of interest

The author declared no conflict of interest.

## Ethical approval

Victoria University Human Research Ethics Committee provided ethical approval for this study (HRE19–019).

## Informed consent

Informed consent was obtained from all individual participants included in the study.

## Availability of data

The datasets analyzed for the current study are available upon reasonable request.
